# Interdisciplinary Management of a Class II Bimaxillary Protrusion Case With a Traumatized Maxillary Central Incisor: A Case Report

**DOI:** 10.7759/cureus.73822

**Published:** 2024-11-16

**Authors:** Garima Arora, Priyanka Mehta, Sana Bint Aziz, Puneet Batra, Ashish K Singh

**Affiliations:** 1 Department of Orthodontics and Dentofacial Orthopedics, Manav Rachna Dental College, Faridabad, IND

**Keywords:** bimaxillary protrusion, class ii malocclusion, interdisciplinary treatment, orthodontic camouflage, traumatized incisor

## Abstract

The class II bimaxillary protrusion malocclusion with class II or end on molar relation is generally associated with procumbency of lips. This case report presents a case of a 17-year-old nongrowing male patient with a traumatized left central incisor due to a fall with a chief complaint of forwardly placed and gap in upper front teeth. The traumatized left central incisor with Ellis class IV fracture was with a loss of crown structure and a poor prognosis of remaining tooth structure. Class II bimaxillary protrusion malocclusion was planned to be treated with orthodontic camouflage. A fixed mechanotherapy orthodontic treatment was performed by extracting the right first premolar and left central incisor and substituting the extracted maxillary central incisor with the lateral incisor. Thus, the maxillary canine was substituted for the lateral incisor, and the first premolar was substituted for canines. It was followed by substituting the lateral incisor with canine by recontouring the crown and restoring the substituted lateral incisor with veneer. The case was completed in 24 months, smile esthetics were improved, and functional occlusion of the class I canine and Angle's class II molar relationship bilaterally was achieved.

## Introduction

Class II malocclusion is a common malocclusion with a global prevalence of 19.56% in permanent dentition [1]. Class II skeletal base is characterized by a retrusive mandible position relative to the maxilla or protrusive maxillary anterior teeth, resulting in a convex facial profile and an increased overjet [1]. The global prevalence of increased overjet was found to be 20.14%. Various etiological factors are associated with class II malocclusion, such as genetic factors, ethnicity, and environmental. Class II malocclusion has been reported to be associated with decreased masticatory efficiency, speech difficulties, and increased risk of dental trauma. The most prevalent class II malocclusion is class II division 1 [2]. The maxillary central incisors, due to their prominent position in the dental arch, are especially vulnerable to traumatic injuries in individuals with a class II relationship, where the increased overjet predisposes these teeth to impact injuries, fractures, and luxations [2,3]. The global prevalence of traumatic dental injuries in permanent dentition was found to be 15.2% [2]. Trauma to the maxillary central incisors can complicate the clinical scenario, making the management of class II malocclusion even more challenging and requiring a multifaceted treatment approach [3,4].

Dental trauma to the maxillary central incisors can have significant consequences for the dental pulp, periodontal tissues, and supporting structures, often resulting in a range of injuries, such as crown fractures, root fractures, and pulp necrosis, leading to poor prognosis [5]. The clinical management of such cases goes beyond simple orthodontic correction and frequently demands interdisciplinary treatment. Interdisciplinary treatment planning plays a crucial role in addressing complex dental cases, as it combines the expertise of different specialties to develop a comprehensive treatment strategy [4]. Orthodontic treatment aims to establish a stable occlusion by correcting the malocclusion and aligning the dental arches. At the same time, restorative procedures may involve direct or indirect restorations, vital pulp therapy, and nonvital pulp therapy, such as root canal treatment or even surgical intervention, depending on the severity of the trauma [6].

This case report discusses the interdisciplinary management of a patient presenting with a class II bimaxillary protrusion malocclusion and a traumatized maxillary central incisor. The treatment involved a coordinated approach between orthodontic alignment and restorative procedures to contour and restore tooth structure. This case report highlights the significance of proper diagnosis, treatment planning, timely intervention, and other disciplines in managing such complex cases. The case illustrates the successful resolution of a challenging clinical scenario and emphasizes the value of interdisciplinary collaboration in delivering patient-centered, functional, and aesthetic outcomes.

## Case presentation

Diagnosis and etiology

**Figure 1:**
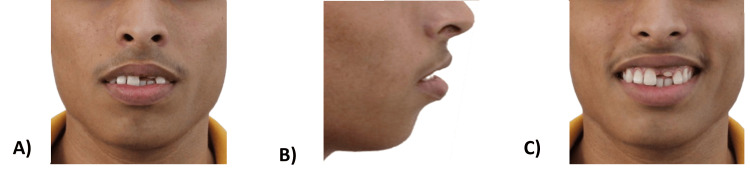
Pretreatment extraoral photographs: (A) Frontal view at rest. (B) Profile view. (C) Frontal view while smiling.

A 17-year-old nongrowing male patient reported to the Department of Orthodontics and Dentofacial Orthopedics of Manav Rachna Dental College, Faridabad, with the chief complaint of forwardly placed upper front teeth and gaps in upper front teeth. He revealed no relevant medical history and dental history of trauma to his upper front teeth eight years ago, which was not restored. Upon extraoral examination at rest, he presented with a grossly symmetrical face of leptoprosopic facial form with potentially competent lips. He had a convex facial profile, average nasolabial angle, posterior divergence, average mentolabial sulcus, flat forehead, and average nasofrontal angle, with a straight nasal bridge and rounded nasal tip. The patient had an average mandibular plane angle with a protruding lower lip. On smile analysis, the patient had a nonconsonant smile, cuspid smile style, and high lip line with average buccal corridor width. Facial proportions revealed proportionate horizontal fifths and increased lower anterior facial height (Figure 1).

**Figure 2:**
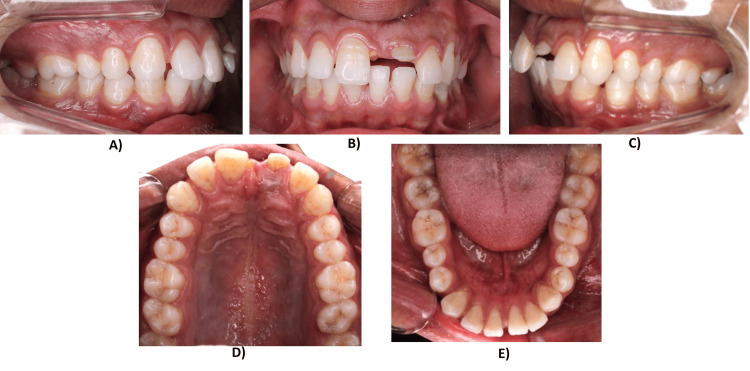
Pretreatment intraoral photographs: (A) Right buccal view. (B) Frontal view. (C) Left buccal view. (D) Maxillary occlusal view. (E) Mandibular occlusal view.

On intraoral examination, the patient presented with a full complement of permanent dentition and traumatized 21 with Ellis class IV (nonvital pulp with loss of crown structure) fracture due to trauma. The canine relationship was end-on on the left side and had an end-on class I molar relationship bilaterally on the right side. Both upper and lower arches are U-shaped. The upper and lower incisors are proclined. There is spacing present in both the upper and lower arches. Additionally, there is an increased overjet and overbite. Also, the lower midline was shifted to the left side by 1 mm in relation to the facial midline, and the upper midline could not be assessed (Figure 2). Functional examination revealed a normal pattern of speech. However, due to the missing upper left central incisor, fricative sounds, such as s, z, th, and zh, and labiodental sounds, such as f and v, were affected. The patient presented oronasal respiration, normal swallowing pattern, and no evidence of temporomandibular joint disorder, with a mouth opening of about 54 mm.

**Figure 3:**
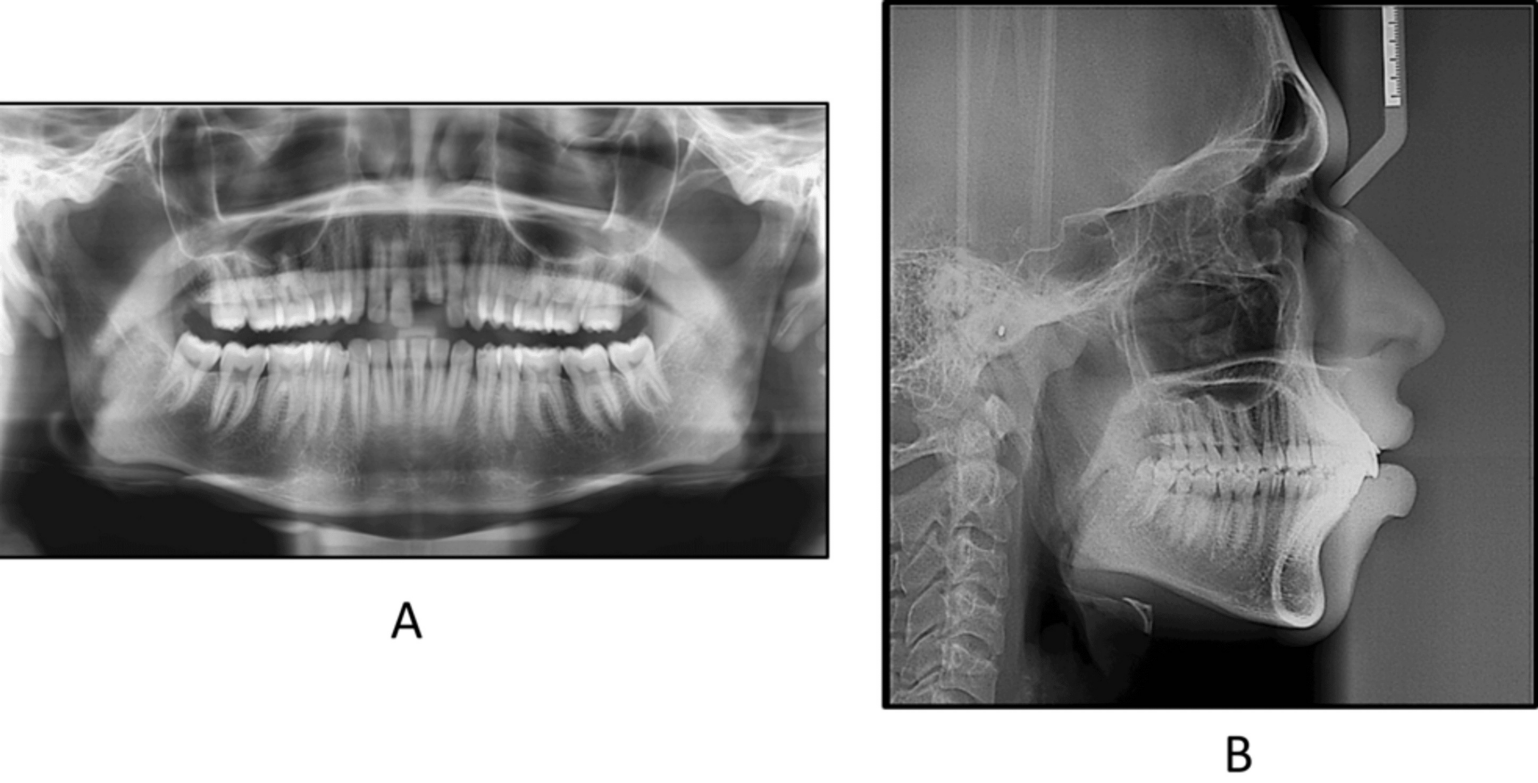
Pretreatment (A) panoramic view and (B) lateral cephalogram

An examination of the orthopantomogram radiograph (Figure 3A) revealed a root stump with a small crown structure with respect to 21 and fully developed and erupted third molars. The lateral cephalogram (Figure 3B) examination revealed the patient was in cervical vertebra maturation index stage 6, i.e., maturation stage, with no remaining skeletal growth. There was a skeletal class II maxillomandibular base relation due to the forwardly placed maxilla (angle between sella, nasion, and point A = 84°) and normally placed mandible (angle between sella, nasion, and point B, SNB = 78°), leading to a discrepancy in jaw base relationship (angle between point A, nasion, and point B = 6°). The Wits appraisal revealed a discrepancy of 2 mm, and the Beta angle was 18°, indicating a class II malocclusion.

Cast evaluation revealed a class II (end-on) molar and class I canine on the right side and end-on relation on the left side; intermolar width of 44 and 38 mm in the upper arch and lower arch, and intercanine width of 30 and 21 mm in the upper and the lower arch, respectively; the curve of Spee of 1 mm in the lower arch, and overjet and overbite of 4 and 4 mm, respectively.

Furthermore, the cephalometric findings revealed lower anterior facial height, i.e., 66 mm, Jarabak's ratio of 54%, a Frankfort-mandibular plane angle of 22°, and a line from sella to nasion (SN) gonion-gnathion (GoGn) of 31°, suggesting an average growth pattern. The dental analysis indicated that the maxillary incisors are proclined at an angle of 30° to the nasion (UI-NA) and 130° to the Sella-Nasion (UI-SN). Additionally, the mandibular incisors are also proclined at 30° to the nasion (LI-NB) and at an IMPA of 105°. The soft tissue analysis showed a protuberance of upper and lower lips and increased upper lip strain (Table 1).

**Table 1:** Cephalometric findings N perp: nasion perpendicular; A: supraspinale; B: subspinale; GoGn: gonion-gnathion; Pog: pogonion; Y-axis: growth axis; LAFH: lower anterior facial height; FMA: Frankfurt mandibular angle; IMPA: incisor mandibular plane angle; E-line: esthetic line; S-line: Steiner's line; H-angle: Holdaway's angle; ANB: angle between point A, nasion, and point B; SN: line from sella to nasion; SNA: angle between sella, nasion, and point A; SNB: angle between sella, nasion, and point B; U1 to NA: angle between upper incisor to nasion A point line; U1 to SN: angle between upper incisor to sella nasion line; U1 to A-Pog: linear distance from upper incisor to point A pogonion line; L1 to A-Pog: distance between the edge of the lower incisor and the A-Pog line

Measurements	Norms	Pretreatment	Posttreatment
SNA (°)	82	84	82.5
N perp to point A (mm)	0-1	1	0
Effective maxillary length (mm)	-	86	86
SNB (°)	80	78	79
N perp to Pog (mm)	-	-8	-6
Effective mandibular length (mm)	113-116	110	110
Facial angle (°)	88	85	87
ANB (°)	2	6	3.5
Wits appraisal (mm)	-1	4	2
Maxillomandibular differential (mm)	-	14	14
FMA (°)	22	22	24
SN-GoGn (°)	32	31	32
Y-axis (°)	59.4	59	60
Facial axis (°)	0 ± 3.5	-3	-3.5
LAFH (mm)	63-64	66	67
U1 to NA (mm)	4	9	2
U1 to NA (°)	22	30	21
U1 to A-Pog (mm)	2.7	3	3
U1 to SN (°)	104	115	107
L1 to NB (mm)	4	7	4
L1 to NB (°)	25	30	25
IMPA (°)	90	105	100
L1 to A-Pog (mm)	1-3	-2	2
Overbite (mm)	1-2	4	3
Overjet (mm)	1-3	4	2
Interincisal angle (°)	135.4	107	125
Nasolabial angle (°)	102 ± 8	108	110
Upper pharynx (mm)	15-20	12	14
Lower pharynx (mm)	11-14	9	10
Skeletal profile (°)	177.5	165	160
Soft tissue profile (°)	158.73	152	151
Total profile (°)	135.23	127	127
Length of upper lip (mm)	23	18	18
Length of lower lip (mm)	35.9	40	40
Upper lip protrusion (mm)	4.72 ± 1.70	4	2
Lower lip protrusion (mm)	2.83 ± 1.64	+3	+1
Upper lip to E-line (mm)	-0.26	0	0
Lower lip to E-line (mm)	2.15	4	2
Upper lip to S-line (mm)	-0.26	-1	-1
Lower lip to S-line (mm)	2.15	-3	-1
H-angle (°)	M = 14.30; F = 16.68	13	10
Upper lip strain (mm)	0-1	4	3

Treatment objectives and planning

The primary treatment objectives were to correct the inclination and protuberance of the maxillary anterior, restore the normal appearance of the maxillary anterior teeth, and correct the occlusion. Ideal overjet and overbite relationships were desirable to establish proper anterior guidance. Additional objectives were to achieve upper dental midline coinciding with facial midline and to achieve class II molar relation bilaterally. The case was planned to be treated with class II camouflage treatment. The case was treated as an extraction case with extraction of 21 and 14, fixed orthodontic mechanotherapy. The retention plan was with maxillary and mandibular bonded flexible spiral wire retainers.

Treatment progress

A written informed consent was obtained from the patient to take records and use them for research before commencing the treatment. An oral surgeon extracted 14 and 21. After a week of healing, a preadjusted edgewise appliance with a McLaughlin, Bennet, and Trevisi prescription (0.022 x 0.028" slot) was bonded in both arches. The left maxillary central incisor bracket was placed on the left lateral incisor to allow a more palatal root torque and reduce mesial inclination. The lateral incisor bracket was bonded to the canine to decrease its buccal root torque. The canine bracket was bonded on the first bicuspid to consider the canine substitution. Second molars were bonded to as act as a mechanics of bite opening.

**Figure 4:**
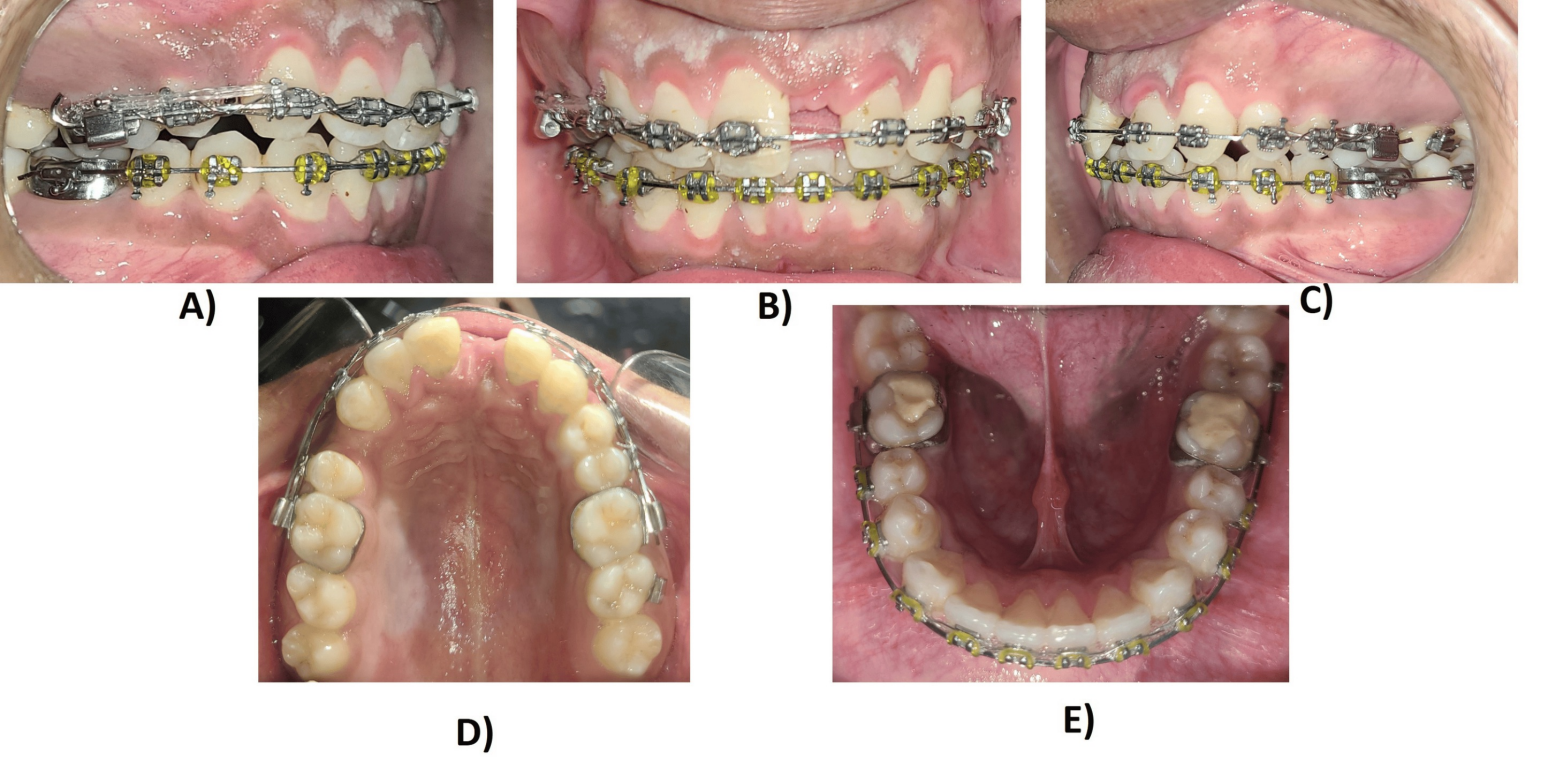
Treatment progress: mesialization of lateral incisor and space closure: (A) Right buccal view. (B) Frontal view. (C) Left buccal view. (D) Maxillary occlusal view. (E) Mandibular occlusal view.

Leveling and alignment were done using a sequence of 0.014", 0.016", and 0.018" round NiTi archwires for four months. After leveling and alignment, space closure was done using frictional/sliding mechanics. The space closure was carried out at 0.019 x 0.025" stainless steel archwires. To compensate for the play in 0.019 x 0.025" wire, an additional torque of 10° was incorporated. Burning of molar anchorage was desirable bilaterally to achieve class II molar relation. Mesialization of 22 was done to substitute it as a central incisor (Figure 4).

The interdisciplinary discussion had been ongoing during the end stages of orthodontic treatment. Before removing the appliance, the patient was referred to the periodontics department for a gingivectomy. Before proceeding with the gingivectomy, thorough scaling and polishing were done, and we waited for two weeks postscaling for the healing of the gingiva. After the residual swelling had subsided, the gingivectomy was carried out by a periodontist. The brackets were removed when the final position of the lateral incisor, i.e., 22 at the place of 21, had been achieved for an esthetically and functionally desirable veneer at that position. Subsequently, the patient was referred to the prosthodontics department for crown preparation and veneer. As retention, maxillary and mandibular bonded lingual retainers were placed.

Treatment results

**Figure 5:**
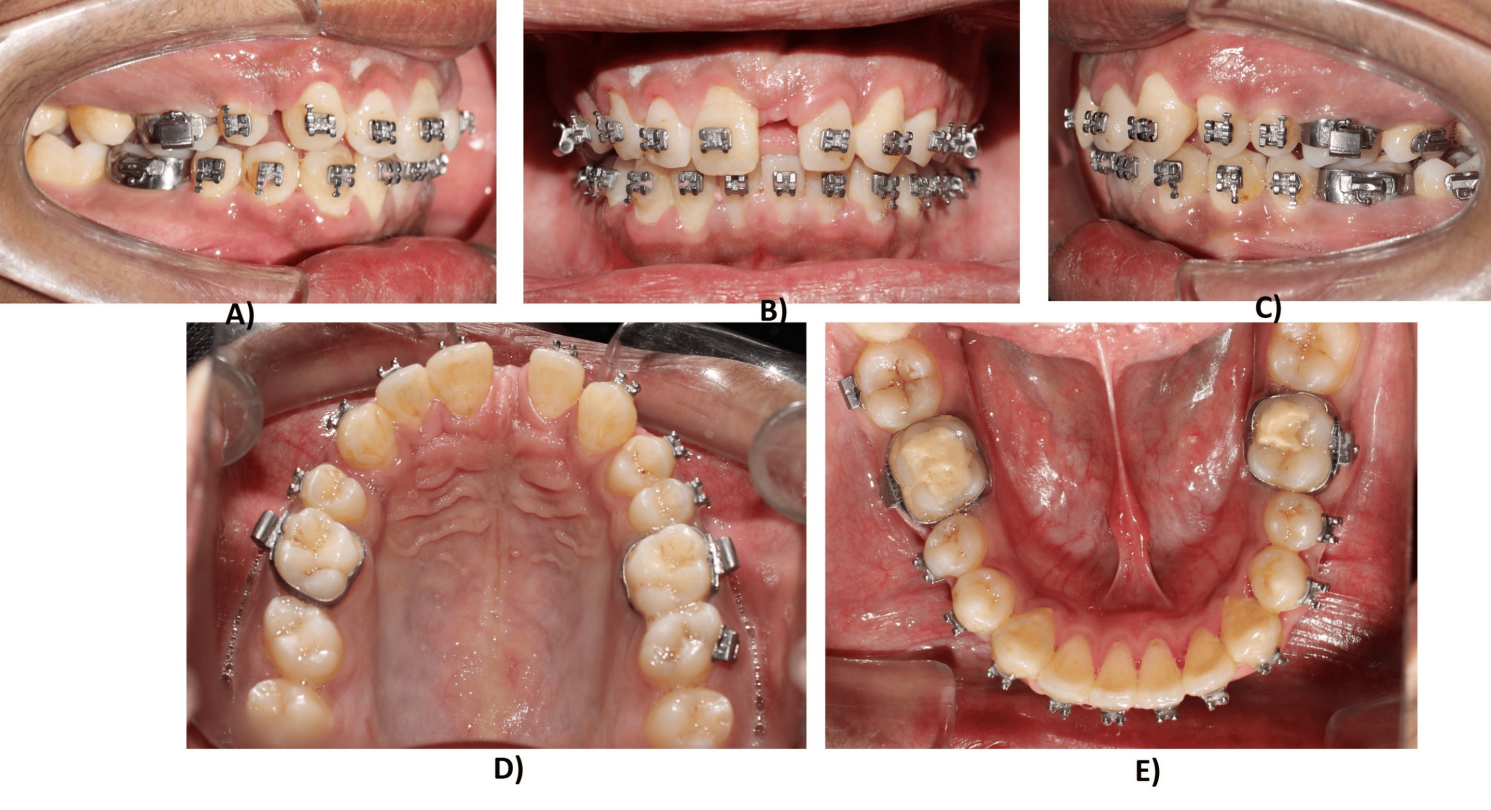
Mid-treatment intraoral photographs: (A) Right buccal view. (B) Frontal view. (C) Left buccal view. (D) Maxillary occlusal view. (E) Mandibular occlusal view.

**Figure 6:**
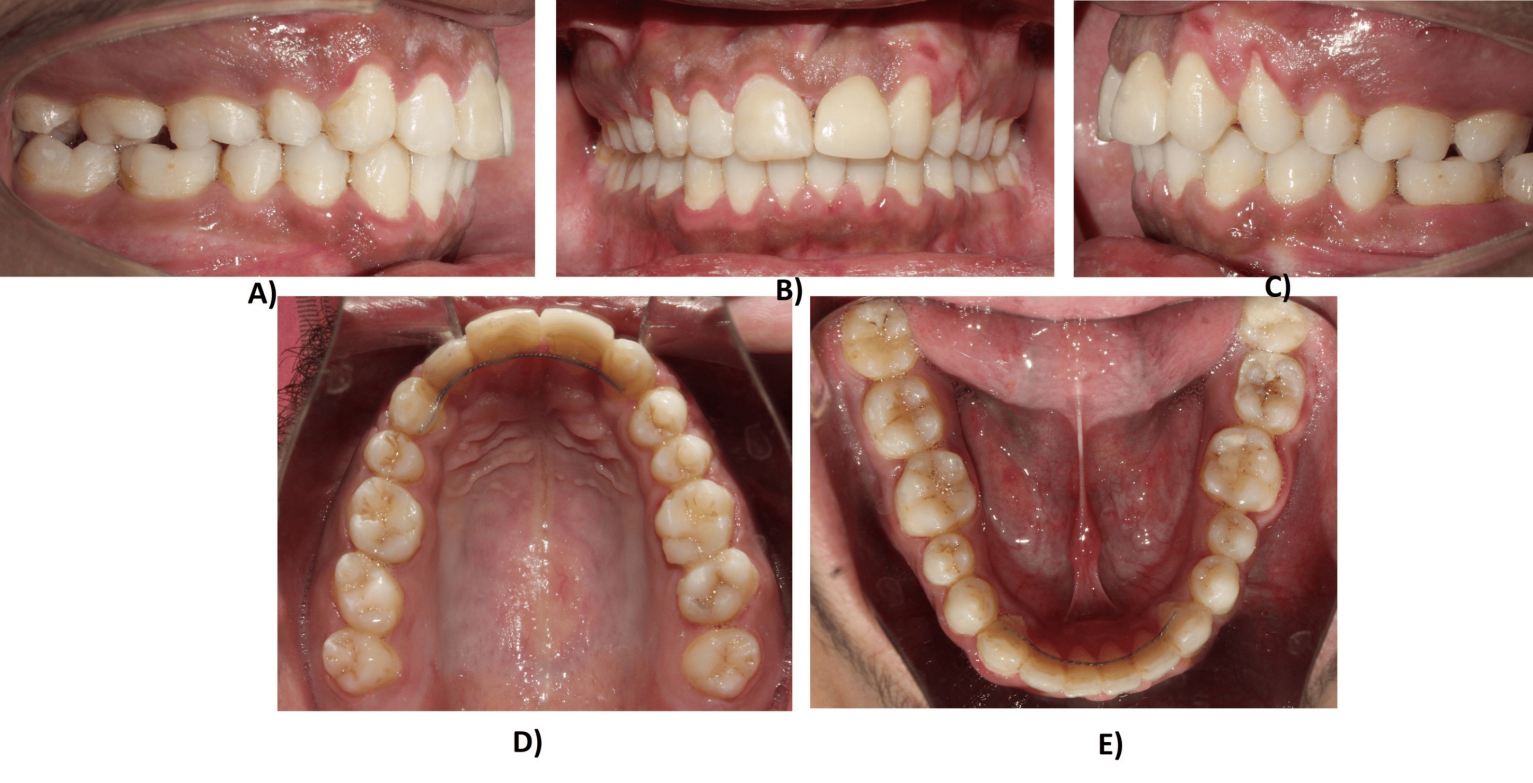
Posttreatment intraoral photographs with bonded lingual retainers: (A) Right buccal view. (B) Frontal view. (C) Left buccal view. (D) Maxillary occlusal view. (E) Mandibular occlusal view.

**Figure 7:**
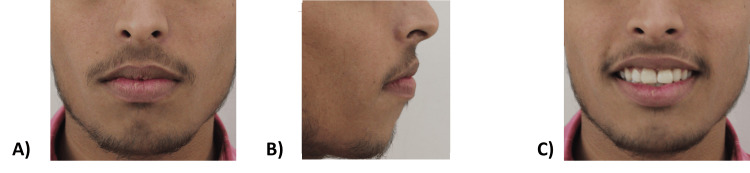
Posttreatment extraoral photographs: (A) Frontal view at rest. (B) Profile view. (C) Frontal view while smiling.

The treatment was completed in 24 months after the prosthetic restoration of the central incisor. By the end, we achieved good functional occlusion and improved the soft profile of the patient. The final molar relationship was Angle's class II cusp to fossa relationship and satisfactory canine relation. An overjet and overbite of 2 mm were also achieved with matching upper and lower midlines along with a harmonious soft tissue profile. The canine was re-contoured into a lateral incisor, and the restoration of the substituted lateral incisor using a veneer was achieved. Space closure in the lower arch was achieved (Figures 5-7).

**Figure 8:**
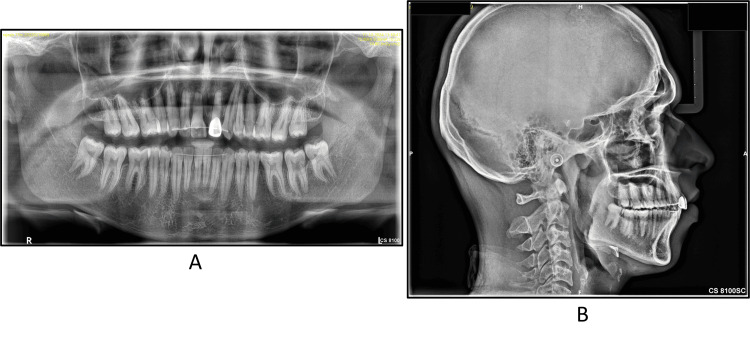
Posttreatment (A) OPG and (B) lateral cephalogram OPG: orthopantomogram

**Figure 9:**
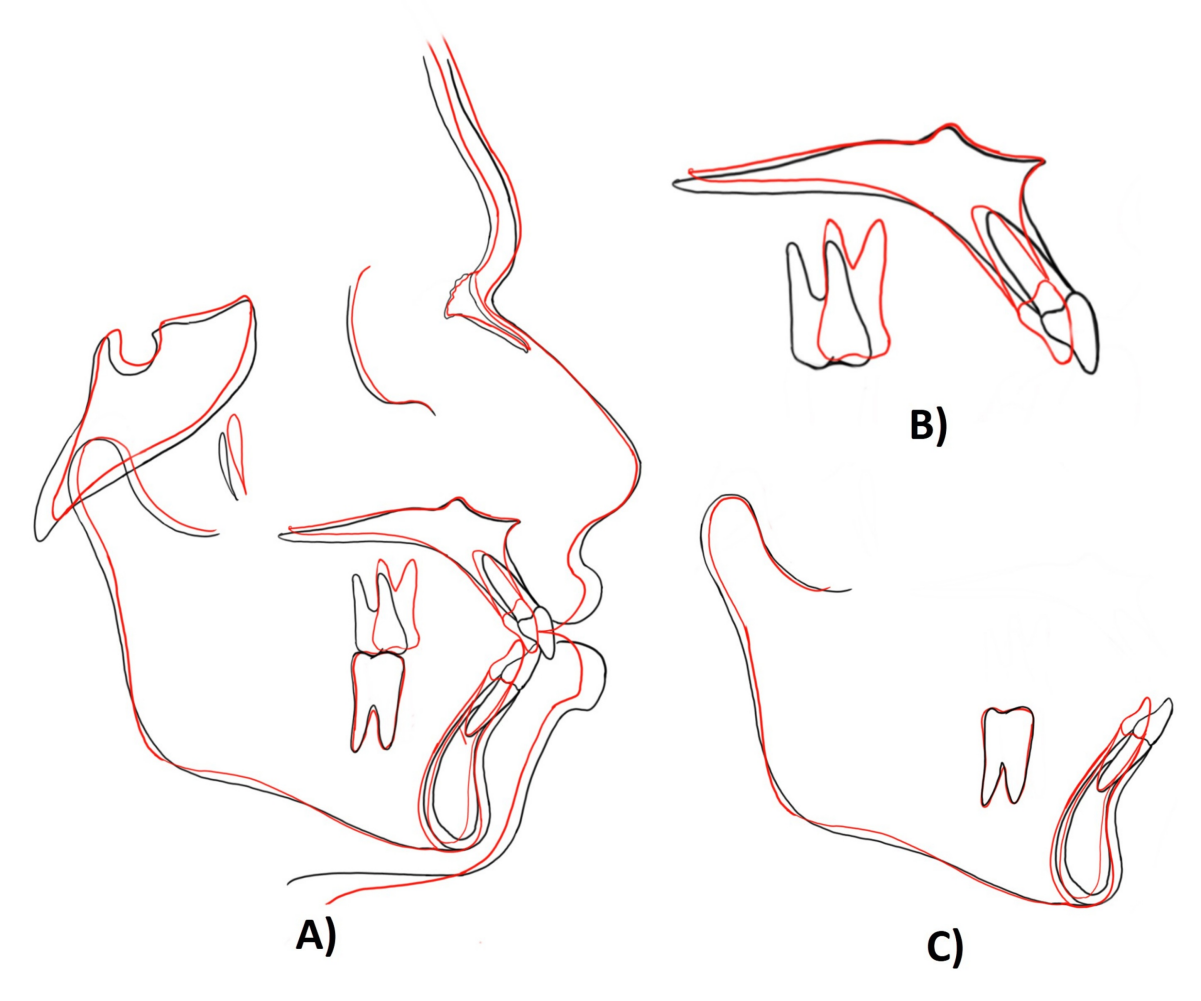
Pretreatment (black) and posttreatment (red) superimpositions. (A) Superimposition at SN. (B) Regional superimposition of maxilla. (C) Regional superimposition of mandible SN: line from sella to nasion

The posttreatment panoramic view depicted root parallelism at the end of the treatment (Figure 8A). The posttreatment cephalogram revealed an improvement in the maxillomandibular incisor relationship with desirable upper and lower incisors inclination, i.e., 7° in the upper and 5° in the lower. The angle of convexity also improved by 3°. The Wits appraisal revealed a reduced value of 2 mm from the pretreatment value of 4 mm. The IMPA was reduced from 105° to 100°, which was desirable. As soon as we bond the maxillary teeth, it disrupts the occlusion, determining a slight forward position of the mandible, and an increase in the SNB angle by 1° is obtained during treatment. This correction in incisor inclination and mesialization of maxillary molars to attain a class II relationship can be well appreciated in the superimpositions (Figures 8B, 9).

## Discussion

The successful orthodontic treatment of a class II bimaxillary case involving a traumatic central incisor presents a unique set of challenges. Class II malocclusions are commonly characterized by a retrusive mandible, protrusive maxilla, or a combination [7]. According to McNamara's analysis, if A point is ahead of the Nasion perpendicular, it shows maxillary prognathism [8]. In this case report, we analyzed the maxillary skeletal parameters and found that the class II skeletal base was due to the prognathic maxilla. Class II malocclusion can often be treated by orthodontic camouflage or orthognathic surgery, depending on the severity of skeletal sagittal dysplasia [7]. In this case, orthodontic camouflage was used to correct malocclusion using fixed orthodontic appliances.

The traumatized central incisor with Ellis class IV fracture presented a challenge in its management. Traumatized teeth are prone to complications like pulp necrosis, root resorption, and even ankylosis. In the literature, it is stated that dental trauma occurs more often in adolescent boys than in girls [9]. The maxillary central incisors are most commonly affected (84%), followed by the mandibular central incisors (7.5%), maxillary lateral incisors (4.5%), and maxillary canines (3%) [9]. Proper assessment and pretreatment of the incisor's endodontic status are essential before orthodontic forces can be applied. According to some studies, traumatized teeth can respond favorably to orthodontic movement if the forces are controlled and the tooth's vitality is carefully monitored [10,11]. In this case, the endodontic treatment of the central incisor was not possible due to its poor prognosis, and preventing any adverse effects during treatment, the extraction for that central incisor was planned.

In case of substitution, there are a few factors that need to be considered, such as parallelling the roots of lateral incisors, reducing the root prominence of the canine by placing the bracket of the lateral incisor on the canine to create a lingual torque, rotating the first premolars slightly in the mesiopalatal direction, and reducing palatal cusp to resemble canine [12-14]. In this case, we considered all these factors. However, the ideal correction of substituted canine torque was not achieved. A gingival cleft is well appreciated in posttreatment left lateral intraoral view (Figure 8).

This patient's orthodontic management involved using fixed appliances to align the maxillary and mandibular arches, close any residual spaces, and achieve the class II molar and class I canine relationship. The orthodontic approach of substituting lateral incisors in place of missing or extracted central incisors has been implemented [14]. Using elastics helped achieve a favorable anteroposterior relationship and interarch anchorage for space closure. The ideal inclination and position of maxillary and mandibular incisors can be achieved with the help of space-gaining methods such as distalization or extractions, etc. [15,16]. The inclination of maxillary and mandibular incisors was corrected due to space closure in the lower arch and extractions of 14 and 21, followed by space utilization to correct overjet and inclination.

This case highlights the significance of customized treatment planning in patients with a history of dental trauma. The integration of endodontic and orthodontic care ensured that the central incisor maintained its vitality and stability, while the orthodontic treatment resulted in functional and esthetic improvements [17,18]. Managing traumatic dental injuries alongside orthodontic correction necessitates careful monitoring and timely interventions to minimize risks such as root resorption and relapse. The main limitation of our report is that follow-up results are not present to evaluate long-term stability and retention. The long-term follow-up is essential as there is a risk of relapse, and periodontal health maintenance is required.

## Conclusions

This case report demonstrates the successful orthodontic correction of a class II bimaxillary protrusion malocclusion in a patient with a history of trauma to the central incisor. The combination of fixed orthodontic appliances and pretreatment endodontic care enabled the correction of the malocclusion while preserving the health and vitality of the traumatized tooth. Careful application of controlled orthodontic forces was essential in minimizing the risks associated with moving a previously injured tooth. The success of the treatment underscores the importance of interdisciplinary collaboration between orthodontists and endodontists in managing complex cases involving dental trauma.

Long-term retention and follow-up are crucial to ensure the stability of the orthodontic correction and to monitor the long-term prognosis of the treated incisor. This case emphasizes that with proper planning and collaboration, even complicated orthodontic cases involving dental trauma can be successfully managed, resulting in both functional and esthetic outcomes for the patient.
